# Berberine as a Mitochondrial Metabolic Reprogrammer in Metabolic Dysfunction-Associated Steatotic Liver Disease (MASLD): Adaptive Energetic Stress, Quality Control, and Translational Limitations

**DOI:** 10.3390/nu18203366

**Published:** 2026-10-14

**Authors:** Hossein Rafiei, Kosar Omidian, Miguel Tolentino, Ali Jafari, Hossein Bahari, Mousa Khalafi

**Affiliations:** 1 Department of Nutrition and Dietetics, Saint Louis University, Saint Louis, MO 63104, USA; miguel.tolentino@health.slu.edu; 2 College of Pharmacy and Nutrition, University of Saskatchewan, Saskatoon, SK S7N 2Z4, Canada; kosar.omidian@gmail.com; 3 Student Research Committee, Department of Community Nutrition, Faculty of Nutrition Sciences and Food Technology, National Nutrition and Food Technology Research Institute, Shahid Beheshti University of Medical Sciences, Tehran 1467864763, Iran; ajafari.nut@gmail.com; 4 Department of Nutrition, Faculty of Medicine, Mashhad University of Medical Sciences, Mashhad 9919191778, Iran; baharihossein3@gmail.com; 5 Department of Sport Sciences, Faculty of Humanities, University of Kashan, Kashan 8731753153, Iran; mousa.khalafi@yahoo.com

**Keywords:** berberine, metabolic dysfunction-associated steatotic liver disease, nonalcoholic fatty liver disease, mitochondrial function, oxidative phosphorylation, mitochondrial quality control, oxidative stress, sirtuins

## Abstract

Metabolic dysfunction-associated steatotic liver disease (MASLD) is closely linked to mitochondrial dysfunction, including altered oxidative phosphorylation, disrupted fatty-acid oxidation, redox imbalance, abnormal mitochondrial dynamics, and impaired quality control. Berberine (BBR), a plant-derived isoquinoline alkaloid used as a nutraceutical, has been described both as a partial inhibitor of respiratory-chain complex I and as an intervention that improves mitochondrial function. This narrative review critically synthesizes evidence from hepatoma cells, primary hepatocytes, animal models, and human studies to reconcile these divergent effects. The preclinical literature supports a proposed, context-dependent model in which BBR imposes an energetic constraint during nutrient overload while activating AMP-activated protein kinase- and sirtuin-linked pathways that remodel substrate handling, respiration, redox balance, mitochondrial architecture, and autophagy. The strongest mechanistic evidence supports complex I-associated respiratory remodeling, SIRT3-mediated deacetylation of long-chain acyl-CoA dehydrogenase, SIRT1-mediated stabilization of carnitine palmitoyltransferase 1A, and SIRT1-dependent autophagy. Evidence for consistent mitochondrial biogenesis, selective mitophagy, mitochondria–endoplasmic reticulum contact-site remodeling, mitochondrial DNA danger signaling, and ferroptosis regulation in MASLD remains limited. Human evidence is mixed: a recent placebo-controlled trial found no significant reduction in liver fat with pure BBR, and no included trial directly measured hepatic mitochondrial function or established a mitochondria-mediated clinical effect. Therefore, BBR is best viewed as a context-dependent modulator of hepatic mitochondrial metabolism rather than a uniform mitochondrial enhancer. Pure BBR should not be conflated with whole barberry fruit, uncharacterized botanical preparations, or berberine ursodeoxycholate (HTD1801). Translation requires characterized formulations, exposure-matched models, and direct mitochondria-focused endpoints.

## 1. Introduction

Metabolic dysfunction-associated steatotic liver disease (MASLD), formerly termed nonalcoholic fatty liver disease (NAFLD), is the most common form of steatotic liver disease and is characterized by hepatic steatosis in the presence of at least one cardiometabolic risk factor [1]. Earlier global estimates based on NAFLD definitions placed MASLD prevalence at approximately one-third of adults worldwide, whereas more recent estimates approach 38%, and its prevalence continues to increase in parallel with obesity, insulin resistance, and type 2 diabetes [2,3]. The disease encompasses a heterogeneous spectrum ranging from isolated hepatic steatosis to metabolic dysfunction-associated steatohepatitis (MASH), which is characterized by steatosis, hepatocellular injury, and inflammation and may progress to advanced fibrosis, cirrhosis, hepatocellular carcinoma, and liver-related death [4,5,6]. Because most of the mechanistic and clinical studies discussed in this review were published before adoption of the new nomenclature, the terms NAFLD and nonalcoholic steatohepatitis (NASH) are retained only when describing the terminology and diagnostic criteria used in the original studies.

Mitochondria play a central but disease-stage-dependent role in the development and progression of MASLD. Under physiological conditions, hepatic mitochondria oxidize fatty acids through β-oxidation and transfer the resulting reducing equivalents to the electron-transport chain to support oxidative phosphorylation and ATP synthesis. During the early stages of hepatic lipid accumulation, increased fatty-acid delivery may stimulate mitochondrial β-oxidation and respiratory activity as a compensatory adaptation to substrate oversupply. However, persistent lipid overload may exceed mitochondrial oxidative capacity and promote incomplete fatty-acid oxidation, respiratory-chain dysfunction, impaired metabolic flexibility, excessive generation of mitochondrial reactive oxygen species (ROS), and lipid peroxidation [7,8,9]. These abnormalities can amplify endoplasmic-reticulum (ER) stress, inflammatory signaling, hepatocyte injury, and cell-death pathways, thereby contributing to the transition from relatively uncomplicated steatosis to MASH and fibrosis [7,9,10]. Importantly, mitochondrial dysfunction in MASLD should not be viewed solely as a generalized reduction in mitochondrial activity. Rather, it reflects a dynamic and disease-stage-dependent loss of mitochondrial adaptability, including altered oxidative flux, impaired substrate handling, disturbed redox homeostasis, and deterioration of mitochondrial quality-control processes [11,12]. Because mitochondria integrate fatty-acid oxidation, oxidative phosphorylation, redox homeostasis, calcium signaling, organelle quality control, and cell-fate regulation, restoring mitochondrial adaptability represents a biologically plausible therapeutic strategy for MASLD [12,13,14,15].

Berberine (BBR) is a plant-derived quaternary isoquinoline alkaloid present in several medicinal plants, including species of *Berberis* and *Coptis*, and is widely marketed as a nutraceutical and dietary supplement [16,17]. Purified BBR should be distinguished from whole barberry fruit and crude botanical preparations. Barberry fruit, traditionally consumed as zereshk in Persian cuisine, contains anthocyanins, phenolic acids, flavonoids, organic acids, vitamins, and variable amounts of alkaloids, including BBR, within a complex food matrix. Because the evidence reviewed here derives predominantly from purified BBR, its effects should not be extrapolated directly to customary dietary consumption of barberry fruit [18].

Although traditionally used for antimicrobial and antidiarrheal purposes, BBR has subsequently been investigated for potential glucose-lowering, lipid-modulating, antioxidant, and anti-inflammatory effects [16,19]. Preclinical studies indicate that BBR can influence several pathways relevant to MASLD, including AMP-activated protein kinase (AMPK) and sirtuin signaling, hepatic lipid synthesis and oxidation, oxidative-stress responses, autophagy, gut-microbiota composition, and interorgan metabolic communication [20,21,22,23,24,25,26]. Clinical findings are mixed. Some studies and pooled analyses have reported improvements in hepatic fat content, aminotransferases, lipid profiles, and indices of insulin resistance, whereas recent placebo-controlled evidence found no significant reduction in liver fat with pure BBR [27,28,29,30]. Interpretation is further limited by heterogeneity in populations, formulations, treatment duration, comparator interventions, and methodological quality. Moreover, BBR has low and formulation-dependent oral bioavailability because of limited intestinal permeability, transporter-mediated efflux, and extensive intestinal and hepatic metabolism. This pharmacokinetic profile complicates direct extrapolation of micromolar concentrations used in cell experiments to clinically achievable systemic exposures, although intestinal and hepatic tissue exposure may exceed circulating concentrations [17,31].

A central unresolved issue is that BBR has been described both as an inhibitor of mitochondrial respiratory-chain complex I and as an intervention that restores mitochondrial respiration, ATP levels, fatty-acid oxidation, and organelle integrity in metabolically compromised tissues [32,33]. These observations are not necessarily contradictory. Partial inhibition of mitochondrial respiration may impose mild energetic stress that activates AMPK- and sirtuin-associated adaptive signaling, whereas in other experimental settings BBR is associated with improved respiratory control after metabolic dysfunction is established. The direction and magnitude of the effects of BBR on mitochondrial function are therefore likely to depend on dose, exposure duration, tissue and cell type, metabolic state, disease severity, and the mitochondrial endpoint assessed [32,33].

A focused reassessment is needed because apparently opposing mitochondrial respiratory responses are often discussed without distinguishing direct functional measurements from molecular markers or adequately accounting for exposure, dose, and model relevance. Recent target-level studies further refine the proposed mechanisms of BBR action on mitochondrial complex I and Ca^2+^ handling [34,35], while recent placebo-controlled clinical evidence challenges the assumption that pure BBR consistently reduces liver fat in MASLD [30]. This review addresses these gaps by distinguishing directly measured mitochondrial effects, evidence of pathway dependence, indirect molecular markers, and mechanistic hypotheses that still require disease-specific validation.

**Figure 1:**
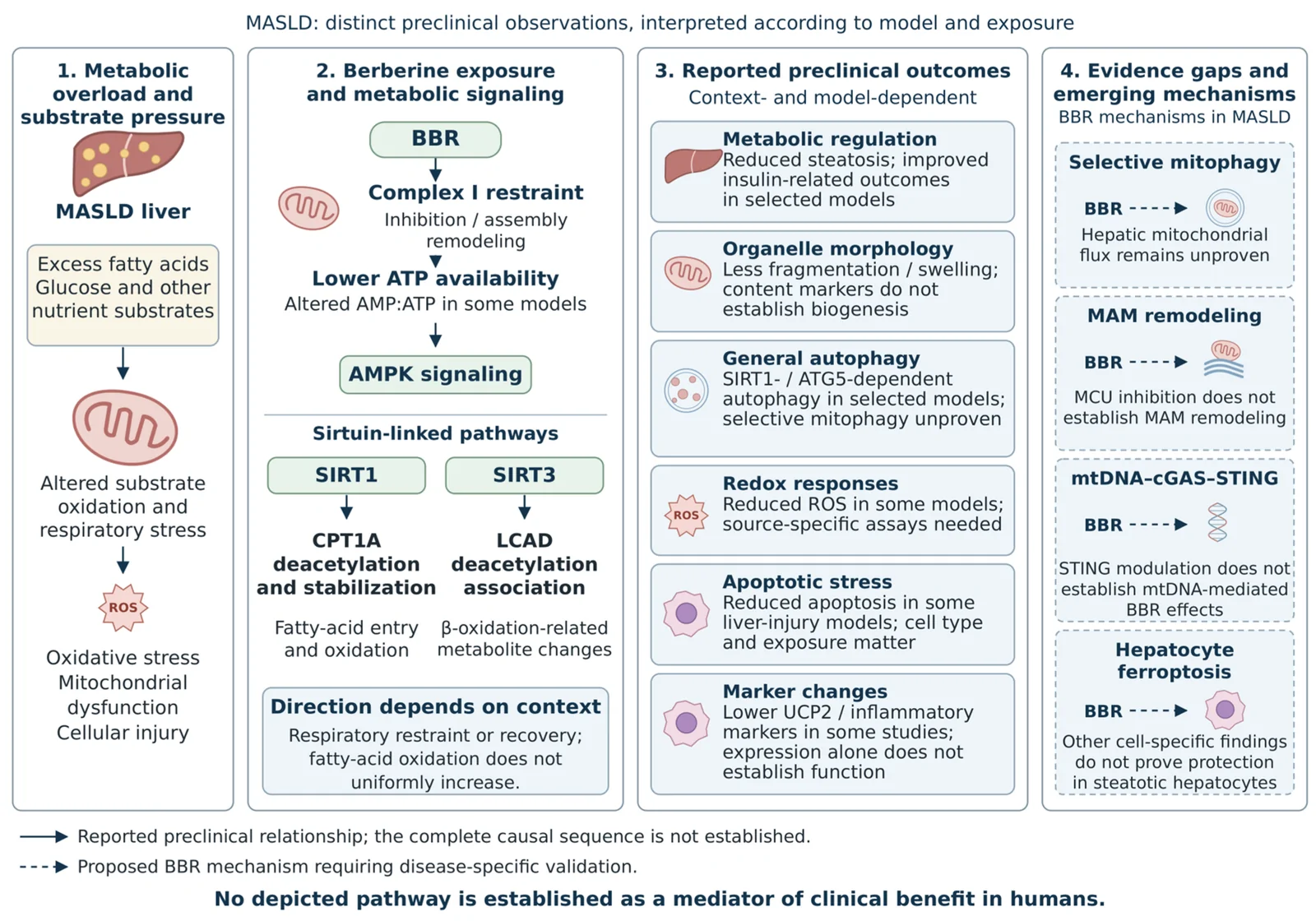
**Proposed context-dependent mitochondrial metabolic reprogramming by berberine in MASLD**.

This narrative review critically evaluates the evidence supporting BBR as a context-dependent mitochondrial metabolic reprogrammer in MASLD. We integrate findings from isolated mitochondria, hepatocyte and hepatoma-cell systems, animal models of diet-induced liver disease, and human clinical studies, with particular attention to respiratory-chain remodeling and energetic stress, AMPK and sirtuin signaling, fatty-acid oxidation and metabolic flexibility, mitochondrial biogenesis and protein acetylation, fusion–fission dynamics, autophagy and mitophagy, membrane potential and proton leak, redox regulation, and mitochondria-dependent cell-death pathways. We further identify emerging but insufficiently investigated mechanisms, including mitochondria-associated endoplasmic-reticulum membranes, mitochondrial calcium handling, mitochondrial DNA damage and danger signaling, and ferroptotic lipid-peroxidation pathways. Finally, we distinguish between mitochondrial mechanisms supported by direct experimental evidence and those inferred from clinical outcomes, because none of the human BBR trials in MASLD included here incorporated direct measurements of hepatic mitochondrial function. The proposed framework describing how BBR may act as a context-dependent modulator of hepatic mitochondrial bioenergetics in MASLD is summarized in Figure 1.

Under conditions of metabolic overload, berberine may impose a partial energetic constraint through respiratory complex I inhibition while activating AMPK-, SIRT1-, and SIRT3-associated pathways that influence fatty-acid metabolism, mitochondrial architecture, general autophagic signaling, redox balance, and cellular injury. Berberine may suppress excessive substrate-driven respiration in some experimental settings while improving respiratory control in mitochondria with established dysfunction. Solid arrows summarize observations or pathway relationships reported in at least one preclinical model; they do not establish that the complete sequence is causal in MASLD. Dashed arrows indicate proposed or insufficiently tested mechanisms. AMPK and sirtuin responses are model-specific, and the schematic does not establish phosphorylation-dependent activation of SIRT1/SIRT3, consistent stimulation of fatty-acid oxidation, increased biogenesis, or selective mitophagy. No depicted pathway has been demonstrated as a mediator of clinical benefit in humans. The strength and direction of these effects may vary according to the model, dose, exposure duration, tissue, and disease stage. Abbreviations: BBR, berberine; CPT1A, carnitine palmitoyltransferase 1A; LCAD, long-chain acyl-CoA dehydrogenase; AMPK, AMP-activated protein kinase; SIRT1, sirtuin 1; SIRT3, sirtuin 3; ROS, reactive oxygen species; MnSOD, manganese superoxide dismutase; UCP2, uncoupling protein 2; TNF-α, tumor necrosis factor alpha; MAMs, mitochondria-associated membranes; mtDNA, mitochondrial DNA; cGAS, cyclic GMP–AMP synthase; STING, stimulator of interferon genes; MASLD, metabolic dysfunction-associated steatotic liver disease.

## 2. Literature Search and Evidence-Appraisal Approach

This is a narrative review. For the revision-stage evidence update on 2 October 2026, targeted retrieval of PubMed/MEDLINE-indexed records and available primary reports was supplemented by examination of references in the manuscript and relevant retrieved reports. Searches combined berberine/BBR with MASLD, NAFLD, MASH, NASH, fatty liver, mitochondrial function, complex I, SIRT3/NDUFS1, biogenesis, mitophagy, calcium uniporter/MCU, mtDNA/cGAS–STING, ferroptosis, clinical trial, adverse events, pharmacokinetics, bioavailability, or formulation. Additional searches used specific study titles and identifiers. No publication-year restriction was applied to this targeted update. It was not an exhaustive database search, and no prospectively registered protocol, duplicate systematic screening, formal risk-of-bias score, or quantitative synthesis was undertaken.

Primary studies were selected for their relevance to BBR exposure, hepatic mitochondrial or mitochondria-related outcomes, or clinical MASLD/NAFLD. Clinical evidence also included a relevant published meta-analysis. Reports on nonhepatic systems, acute liver injury, chemically induced fibrosis, or nonmammalian species were retained selectively to delimit mechanisms and model relevance. Findings with derivatives, ionic salts, mixed formulations, or botanical preparations were not attributed to pure BBR. Review articles informed background and citation identification but were not treated as independent experimental confirmation. Unverifiable mechanistic details were not used to upgrade evidence strength. Table 1 prioritizes representative functional measurements, pathway perturbations, and divergent responses; Table 2 summarizes the principal clinical evidence discussed here and identifies formulation and safety. Neither table is exhaustive.

Evidence was appraised on two separate dimensions: directness of the measurement and relevance of the experimental model. Direct functional evidence requires an appropriate mitochondrial assay, such as substrate-defined respiration, ATP-production rate, coupling/proton leak, membrane potential with suitable controls, calcium handling, or substrate-oxidation flux. ATP content is a steady-state pool, not a production rate. Mechanistic/causal evidence requires target interrogation or pathway perturbation, preferably supported by complementary genetic, pharmacological, or rescue approaches; inhibitor-only and whole-body-knockout studies do not establish hepatocyte-specific causality. Expression, phosphorylation, acetylation, morphology, metabolite profiles, and organelle-content markers alone are indirect/supportive. Extrapolation from other tissues or disease models remains hypothesis-generating for MASLD. These overlapping categories, summarized in Table 3, are interpretive rather than a validated grading system. Replication, controls, exposure, cell type, biological sex, disease stage, and study limitations inform the comparative language used in this review. Studies involving the present authors were assessed using the same criteria as independent reports and were not assigned additional weight because of authorship.

## 3. Adaptive Energetic Stress and Mitochondrial Respiratory-Chain Remodeling

Berberine does not uniformly stimulate mitochondrial activity. One of its best-characterized direct mitochondrial actions is partial inhibition of electron-transport-chain complex I, which can reduce oxidative phosphorylation and alter cellular adenine-nucleotide balance. In several experimental systems, this mild energetic stress has been associated with activation of AMP-activated protein kinase (AMPK), suppression of anabolic metabolism, and improved glucose and lipid handling [36]. However, complex I inhibition, AMPK activation, and the metabolic effects of BBR should not be regarded as interchangeable. In Yu et al., pharmacological AMPK inhibition had little effect on BBR-associated reductions in hepatocyte lipid accumulation, while BBR reduced oxygen consumption, fatty-acid uptake, and β-oxidation-related activity, suggesting that at least some lipid-lowering actions may occur independently of, or in parallel with, AMPK signaling [32]. Yu et al. examined this mechanism in C57BL/6J mice during long-term high-fat-diet (HFD) feeding, incorporating BBR into the diet at a concentration of 1.4 g/kg (a dietary concentration, not a body-weight dose). BBR selectively reduced complex I-dependent oxygen consumption and ATP synthesis in isolated liver and intestinal mitochondria, with little or no effect on complexes II, III, and IV. These changes were accompanied by reduced hepatic fatty-acid uptake, lower expression of lipogenesis- and fatty-acid-transport-related proteins, decreased CPT1A expression, reduced mitochondrial fatty-acid transport and β-oxidation-related activity, and attenuation of hepatic triglyceride accumulation and steatosis. BBR also reduced mitochondrial swelling, increased mitochondrial length, and decreased TEM-based mitochondrial density, which the authors interpreted as a reduction in the number of liver mitochondria. Hepatic mtDNA copy number and citrate synthase activity were also reduced. Collectively, these findings support suppression and remodeling of mitochondrial oxidative metabolism rather than stimulation of mitochondrial biogenesis [32].

The same study also showed that BBR and the direct AMPK activator A769662 both increased AMPK signaling and reduced lipid accumulation in primary hepatocytes but exerted opposite effects on cellular oxygen consumption: BBR decreased respiration, whereas A769662 increased it [32]. This divergence indicates that the respiratory effects of BBR cannot be attributed solely to AMPK activation. Instead, inhibition of complex I and the resulting reduction in mitochondrial substrate oxidation may contribute independently to the ability of BBR to limit lipid accumulation under conditions of nutrient oversupply.

Recent target-level evidence adds a distinct explanation for complex I restraint. Wang et al. reported mitochondrial BBR localization and SIRT3-dependent NDUFS1 deacetylation associated with reversible complex I dissociation and reduced hepatocellular oxidative phosphorylation [34]. This mechanism concerns complex I assembly/abundance rather than direct inhibition of its catalytic enzyme activity. It does not establish universal hepatocyte protection, human target engagement, or improved mitochondrial capacity. SIRT3-linked remodeling can accompany respiratory restraint as well as the functional restoration reported in other models.

Conversely, other studies have reported restoration rather than suppression of mitochondrial respiratory function. In HFD-fed rats with established hepatic mitochondrial dysfunction, Teodoro et al. found that BBR improved mitochondrial membrane potential, oxygen consumption, ATP levels, calcium-retention capacity, and overall oxidative-phosphorylation efficiency [33]. These improvements were associated with increased mitochondrial SIRT3 activity, although the study did not establish a causal or exclusive role for SIRT3 in the recovery of mitochondrial function.

The contrasting findings between Yu et al. and Teodoro et al. should not be explained simply as acute inhibition followed by chronic restoration, because both studies involved sustained BBR exposure. A more plausible interpretation is that the mitochondrial response to BBR depends on the experimental model, baseline bioenergetic phenotype, dose and route of administration, duration of exposure, tissue examined, and the specific mitochondrial endpoints assessed.

Findings from a study by Rafiei et al. in HepG2 cells further support this context-dependent interpretation [21]. In oleic-acid-loaded HepG2 cells, 10 µM BBR reduced steatosis and preserved cellular morphology while increasing SIRT1 activity and AMPKα Thr172 phosphorylation and promoting deacetylation of peroxisome proliferator-activated receptor gamma coactivator 1-alpha (PGC-1α). However, BBR did not restore the oleic-acid-induced reduction in mitochondrial membrane potential and further decreased ATP levels under galactose-supported conditions that increased cellular dependence on mitochondrial oxidative metabolism. Thus, the anti-steatotic effects of BBR occurred despite incomplete recovery of mitochondrial membrane potential and reduced cellular ATP content under mitochondria-dependent conditions, supporting energetic constraint or metabolic reprogramming rather than uniform enhancement of mitochondrial function [21].

Overall, BBR may suppress excessive substrate-driven respiration in some settings while improving respiratory control and ATP-generating capacity in mitochondria with established functional impairment. Thus, BBR is better characterized as a context-dependent modulator of mitochondrial bioenergetics rather than a uniform inhibitor or enhancer of oxidative phosphorylation.

## 4. Mitochondrial Biogenesis and Sirtuin-Dependent Protein Deacetylation

Mitochondrial biogenesis requires coordinated mtDNA replication, expression and import of mitochondrial proteins, membrane expansion, and increased functional mitochondrial content [37,38]. Increased PGC-1α expression therefore indicates activation of a biogenesis-associated transcriptional program but does not, by itself, demonstrate increased mitochondrial biogenesis. Confirmation requires complementary measures of mtDNA copy number, mitochondrial protein content or mass, ultrastructure, and respiratory capacity [38].

Evidence that BBR induces hepatic mitochondrial biogenesis in experimental MASLD is currently limited and inconsistent. Yu et al. reported reductions in mtDNA content and citrate synthase activity after BBR treatment despite improvement in hepatic steatosis [32]. These findings demonstrate that BBR can produce metabolic benefits without increasing mitochondrial content and argue against describing mitochondrial biogenesis as an established or universal mechanism underlying the effects of BBR in fatty liver disease.

In an oleic-acid-loaded HepG2 model, Rafiei et al. found that 10 µM BBR preserved MitoTracker Green fluorescence and increased expression of PGC-1α, NRF1, TFAM, and selected nuclear-encoded respiratory-chain subunits. These findings are consistent with activation of a biogenesis-associated transcriptional program and preservation of mitochondria-related markers. However, mtDNA replication, mitochondrial ultrastructure, citrate synthase activity, mitochondrial respiratory function, and ATP-generating capacity were not assessed. Accordingly, the results should be interpreted as evidence of biogenesis-associated signaling and preservation of mitochondrial-content-related fluorescence rather than definitive demonstration of increased functional mitochondrial biogenesis [23].

In a palmitate-loaded HepG2 study by Rafiei et al., BBR similarly reduced steatosis and cellular ROS, partially preserved MitoTracker Green fluorescence, and increased selected nuclear- and mitochondrial-encoded respiratory-chain transcripts. Nevertheless, BBR did not restore mitochondrial membrane potential and did not significantly suppress palmitate-induced iNOS expression. These findings further indicate that preservation of mitochondria-related content markers or respiratory-gene expression does not necessarily imply recovery of mitochondrial bioenergetic function [24].

Support for a possible biogenesis-related effect comes partly from a study by Li et al. in male ICR mice given 20% fructose in their drinking water for 10 weeks. Oral BBR at 50, 100, or 200 mg/kg/day during the final 4 weeks increased hepatic PGC-1α protein expression and LKB1 and AMPK phosphorylation, while improving insulin sensitivity and hepatic glycogen storage [39]. Notably, BBR reduced the fructose-induced elevation in the hepatic AMP/ATP ratio rather than increasing it, consistent with partial normalization of cellular energy status in this model. However, the study did not assess NRF1, TFAM, mtDNA copy number, mitochondrial abundance, citrate synthase activity, mitochondrial ultrastructure, or mitochondrial respiratory function. Moreover, the model primarily reflected fructose-induced insulin resistance and hepatic lipid accumulation rather than histologically characterized MASLD. These findings therefore support activation of the LKB1/AMPK/PGC-1α signaling axis but do not provide direct evidence that BBR increases mitochondrial biogenesis.

In the current BBR literature, sirtuin-dependent protein deacetylation provides more direct mechanistic evidence for modulation of mitochondrial function than the evidence supporting increased mitochondrial biogenesis. SIRT1 is an NAD^+^-dependent deacetylase localized predominantly in the nucleus but also present in the cytoplasm, where it regulates numerous transcriptional and metabolic proteins, including PGC-1α [40,41]. SIRT1-mediated deacetylation of PGC-1α activates transcriptional programs involved in mitochondrial fatty-acid oxidation and metabolic adaptation [41]. In contrast, SIRT3 is localized primarily within the mitochondrial matrix and is the major mitochondrial NAD^+^-dependent deacetylase that regulates enzymes involved in fatty-acid oxidation, the tricarboxylic-acid cycle, oxidative phosphorylation, and antioxidant defense [40,42]. Activation or increased expression of SIRT1 and SIRT3 may therefore improve mitochondrial metabolic function through post-translational regulation without necessarily increasing mitochondrial abundance.

In HFD-fed rats, Zhang et al. reported that hepatic SIRT3 and CPT1A expression, together with AMPK and acetyl-CoA carboxylase (ACC) phosphorylation, were generally reduced by HFD feeding and increased following concurrent administration of BBR at 100 mg/kg/day [25]. However, the reduction in phosphorylated ACC was not significant at week 8, indicating time-dependent variability in this response. Increased ACC phosphorylation should be interpreted as AMPK-associated inhibitory phosphorylation of ACC rather than activation of the enzyme. Inhibition of ACC is expected to reduce malonyl-CoA formation and thereby relieve malonyl-CoA-mediated inhibition of CPT1A, potentially facilitating mitochondrial fatty-acid entry. Nevertheless, the study measured mainly protein abundance and phosphorylation status and did not directly assess SIRT3 activity, malonyl-CoA concentrations, CPT1A activity, or fatty-acid-oxidation flux. Moreover, neither genetic nor pharmacological disruption of SIRT3 or AMPK was performed. The findings therefore demonstrate coordinated regulation of SIRT3-, AMPK-, ACC-, and CPT1A-associated markers but do not establish a direct causal sequence.

Stronger mechanistic evidence was provided by Xu et al., who showed that BBR reversed HFD-induced suppression of hepatic SIRT3 and reduced the hyperacetylation of long-chain acyl-CoA dehydrogenase (LCAD) [43]. These changes were accompanied by alterations in hepatic acylcarnitine profiles consistent with restoration of mitochondrial β-oxidative metabolism. Importantly, the systemic and hepatic metabolic benefits of BBR were markedly attenuated in whole-body Sirt3-knockout mice, and its effect on β-oxidative metabolism was partially abolished. Thus, the metabolic benefits of BBR are mediated, at least in part, through SIRT3. These findings support SIRT3 dependence and implicate LCAD deacetylation, but whole-body Sirt3 deletion does not establish hepatocyte-specific causality or the necessity of LCAD deacetylation itself, and SIRT3-independent mechanisms also contribute. However, acylcarnitine profiling provides an indirect index of β-oxidative metabolism, and the study did not directly measure SIRT3 enzymatic activity or fatty-acid oxidation flux.

A complementary mechanism involving SIRT1 and CPT1A was reported by Wang et al. [44]. In HFD-fed mice and palmitate-loaded hepatocyte models, BBR increased SIRT1 expression and promoted deacetylation of CPT1A at Lys675. This modification reduced ubiquitin-dependent degradation of CPT1A, increased CPT1A protein stability and activity, and promoted fatty-acid oxidation. SIRT1 overexpression reproduced the triglyceride-lowering effect of BBR, whereas SIRT1 knockdown attenuated this response in HepG2 cells. Site-directed mutagenesis and co-immunoprecipitation experiments further supported Lys675 as a functionally relevant CPT1A acetylation site. These findings support a SIRT1–CPT1A mechanism through which BBR may enhance mitochondrial fatty-acid entry and oxidation. However, the direct molecular target through which BBR increases SIRT1 remains unknown, and the site-specific mechanism was not confirmed using hepatocyte-specific Sirt1 disruption or CPT1A-Lys675 mutant animals. This pathway therefore represents post-translational regulation of a metabolic enzyme rather than direct evidence of mitochondrial biogenesis.

Collectively, the available evidence indicates that BBR modulates hepatic mitochondrial metabolism through at least two context-dependent mechanisms: regulation of respiratory-chain activity and cellular energy status, and sirtuin-dependent post-translational regulation of proteins involved in mitochondrial substrate handling. Evidence supporting SIRT3-mediated LCAD deacetylation and SIRT1-mediated stabilization of CPT1A is substantially stronger than evidence that BBR consistently induces mitochondrial biogenesis. Accordingly, increases in PGC-1α or other biogenesis-associated proteins should be interpreted as evidence of activation of a mitochondrial biogenesis-related transcriptional program rather than increased mitochondrial biogenesis unless accompanied by concordant structural, molecular, and functional evidence of increased mitochondrial content and function.

## 5. Fatty-Acid Oxidation and Metabolic Flexibility

Alterations in hepatic fatty-acid oxidation and mitochondrial oxidative metabolism across MASLD are disease-stage- and context-dependent and should not be described as a uniform suppression of β-oxidation. In obesity and early steatosis, increased fatty-acid delivery may stimulate mitochondrial fatty-acid oxidation, tricarboxylic-acid-cycle activity, and respiratory capacity as an adaptive response intended to limit hepatic triglyceride and lipotoxic-metabolite accumulation. With persistent substrate overload and progression toward steatohepatitis and fibrosis, however, mitochondrial oxidative efficiency and adaptability may decline, contributing to incomplete fatty-acid oxidation, accumulation of lipotoxic intermediates, oxidative stress, and loss of metabolic flexibility [45]. Accordingly, an intervention may improve MASLD either by increasing the efficiency or capacity of fatty-acid oxidation when it is impaired, or by reducing fatty-acid delivery, de novo lipogenesis, and mitochondrial substrate pressure when oxidative metabolism is already excessively stimulated.

BBR appears capable of influencing both substrate delivery and mitochondrial fatty-acid utilization. Wang et al. showed that BBR increased SIRT1 expression and promoted SIRT1-dependent deacetylation of CPT1A at Lys675, thereby reducing CPT1A ubiquitination and degradation and increasing its protein stability and activity [44]. Because CPT1A regulates the rate-limiting transport of long-chain fatty acyl groups into mitochondria, these findings provide mechanistic support for enhanced CPT1A-dependent fatty-acid entry and oxidation in lipid-loaded hepatocytes. SIRT1 overexpression reproduced the triglyceride-lowering effect of BBR, whereas SIRT1 knockdown attenuated BBR-induced increases in CPT1A and reductions in triglyceride accumulation. These experiments strengthen the evidence for a causal role of the SIRT1–CPT1A axis, although the site-specific mechanism was established primarily in cultured and transfected cells and was not validated using hepatocyte-specific Sirt1 disruption or CPT1A-Lys675 mutant animals.

A complementary mechanism involves SIRT3-mediated deacetylation of long-chain acyl-CoA dehydrogenase (LCAD). In HFD-fed mice, Xu et al. found that BBR reversed hepatic SIRT3 downregulation, reduced LCAD hyperacetylation without altering total LCAD abundance, and increased hepatic short- and medium-chain acylcarnitines with carbon-chain lengths below C14, a pattern interpreted as partial restoration of mitochondrial β-oxidative metabolism [43]. Importantly, the systemic and hepatic metabolic benefits of BBR were markedly attenuated in whole-body Sirt3-knockout mice, and the BBR-associated changes in LCAD acetylation, acylcarnitine profiles, and FAO-related gene expression were substantially weakened. These findings provide stronger mechanistic evidence for SIRT3 involvement than studies based solely on protein-expression changes. Nevertheless, the study did not directly measure SIRT3 enzymatic activity or fatty-acid-oxidation flux. Because acylcarnitine concentrations are influenced by substrate entry, intermediate production, and downstream utilization, they should be interpreted as indirect indicators of β-oxidative metabolism rather than direct measurements of flux in the absence of stable-isotope tracing.

The findings of Yu et al. demonstrate that BBR does not invariably increase hepatic fatty-acid oxidation. In male C57BL/6J mice receiving BBR incorporated into an HFD at 1.4 g/kg of diet, BBR reversed HFD-associated increases in CPT1A, FABP1, CD36, and SCD1; reduced CPT1/2-dependent respiration, β-ketothiolase activity, and hepatocyte fatty-acid consumption; and normalized HFD-induced hyperactivation of complex I-linked respiration [32]. These changes occurred alongside reductions in hepatic triglyceride accumulation and steatosis. BBR also lowered the elevated ATP content observed in oleic-acid-loaded primary hepatocytes. Because fatty-acid uptake and lipid synthesis were assessed partly through protein-expression and cellular measurements rather than direct in vivo flux analyses, the results are best interpreted as evidence of broad suppression or normalization of hepatic lipid metabolic activity. Nevertheless, the anti-steatotic effects occurred despite reduced β-oxidative activity, challenging the assumption that increased fatty-acid oxidation is necessary for the hepatic benefits of BBR.

The apparently divergent findings across studies may reflect differences in the degree of substrate overload, baseline mitochondrial phenotype, model duration, BBR exposure, and the balance between complete and incomplete fatty-acid oxidation. When mitochondrial fatty-acid oxidation is impaired, SIRT1–CPT1A and SIRT3–LCAD signaling may contribute to restoration of oxidative capacity. Conversely, in the model used by Yu et al. [32] HFD feeding was associated with increased complex I-linked respiratory capacity and increased expression of proteins involved in fatty-acid uptake and oxidation. BBR normalized these responses and reduced substrate-driven oxidative activity. Thus, BBR may modulate fatty-acid utilization according to the prevailing metabolic phenotype rather than uniformly stimulating β-oxidation. This context-dependent interpretation remains a mechanistic synthesis and requires direct experimental confirmation. Future studies incorporating isotope-labeled fatty acids together with measurements of tricarboxylic-acid-cycle flux, ketogenesis, acylcarnitine turnover, ATP synthesis, and respiratory coupling are needed to distinguish increased complete oxidation from the accumulation of partially oxidized intermediates.

Accordingly, therapeutic stimulation of mitochondrial oxidative metabolism should not be assumed to be uniformly beneficial. In advanced steatohepatitis, increasing fatty-acid oxidation or respiratory flux without simultaneously improving electron-transfer efficiency, respiratory coupling, and antioxidant capacity could aggravate oxidative stress, lipid peroxidation, and inflammatory signaling. The desirable therapeutic response may therefore involve restoration of metabolic flexibility and appropriate substrate–demand matching rather than maximal activation of oxidative metabolism [45].

## 6. Mitochondrial Dynamics, Autophagy, and Quality Control

Mitochondrial homeostasis depends not only on respiratory activity but also on coordinated remodeling through fusion, fission, biogenesis, and selective removal of damaged organelles. Fusion can preserve mitochondrial function by facilitating the exchange of membrane components, proteins, and mitochondrial DNA, whereas fission facilitates organelle distribution and can segregate damaged mitochondrial segments. Either excessive fragmentation or excessive fusion can be detrimental; therefore, the biological objective is restoration of dynamic balance rather than promotion of fusion in all circumstances [46].

The strongest liver-specific evidence that BBR modifies mitochondrial dynamics comes from experimental models of hepatic insulin resistance. Xu et al. identified reduced hepatic SIRT1 and OPA1 expression and abnormalities in dynamics-associated proteins in HFD-fed and db/db mice [47]. OPA1 silencing in HepG2 cells increased mitochondrial fragmentation, reduced ATP content and mitochondrial membrane potential, and impaired insulin signaling. SIRT1 overexpression improved these abnormalities in an OPA1-dependent manner. In palmitate-treated HepG2 cells, BBR increased SIRT1 and OPA1 expression and improved ATP content, membrane potential, glucose consumption, and insulin-signaling responses; SIRT1 silencing partially attenuated these effects. In db/db mice, BBR increased hepatic SIRT1 and OPA1, reduced DRP1 expression and mitochondrial fragmentation, and improved hepatic insulin resistance. These findings support an association between BBR, SIRT1–OPA1 signaling, and a less fragmented mitochondrial phenotype. However, measurements of OPA1, MFN1, and DRP1 abundance do not directly quantify fusion or fission rates, and the study did not assess DRP1 recruitment or phosphorylation, OPA1 processing, or real-time fusion–fission events. Moreover, its primary focus was hepatic insulin resistance rather than histologically characterized MASH. It is therefore more accurate to conclude that BBR shifts dynamics-associated signaling and morphology toward a less fragmented phenotype than to claim that it directly promotes fusion or inhibits fission [47].

Yu et al. similarly reported attenuation of HFD-associated mitochondrial swelling, increased mitochondrial length, and reduced TEM-based mitochondrial density following BBR treatment [32]. These ultrastructural changes occurred alongside reductions in hepatic mitochondrial DNA copy number and citrate synthase activity. The resulting phenotype may therefore represent coordinated structural remodeling and reduced mitochondrial abundance rather than conventional stimulation of both fusion and mitochondrial biogenesis.

BBR also influences general hepatic autophagy, which can degrade lipid droplets, protein aggregates, and cytoplasmic organelles. In high-fat, high-sucrose (HFHS)-fed mice, Sun et al. showed that intraperitoneal BBR increased hepatic autophagic flux, reduced hepatic lipid accumulation, and increased hepatic and circulating FGF21 and circulating ketone bodies [48]. Liver-specific Sirt1 deletion abolished or markedly attenuated the autophagic and anti-steatotic responses to BBR. ATG5 knockdown reduced BBR-induced LC3-II accumulation, whereas chloroquine attenuated its lipid-lowering effect in hepatocytes, providing relatively strong evidence that SIRT1- and ATG5-dependent autophagy contributes to regulation of hepatic lipid storage. However, the intraperitoneal route produces an exposure pattern that may not reflect oral supplementation in humans.

He et al. likewise reported increased autophagic flux in HFD-fed mice and fatty-acid-loaded L02 cells [49]. In hepatocytes, BBR increased ERK phosphorylation while suppressing phosphorylation of mTOR and its downstream targets p70S6K and 4E-BP1. Pharmacological ERK inhibition partially reversed these signaling changes and reduced BBR-induced autophagic flux. Together, the Sun and He studies support activation of general hepatic autophagy but do not establish selective mitochondrial autophagy. LC3-II accumulation, ATG5 dependence, and increased autophagic flux cannot determine whether mitochondria, lipid droplets, or other cellular substrates are preferentially degraded.

Direct evidence that BBR activates PINK1/Parkin-dependent mitophagic flux in mammalian MASLD remains insufficient. In a transverse-aortic-constriction model of heart failure, BBR increased PINK1/Parkin-associated signaling, mitochondrial ubiquitination, and mitochondria-containing autophagosomes, whereas PINK1 knockdown reduced Parkin recruitment and attenuated the beneficial effects of BBR [50]. These findings support regulation of PINK1/Parkin-associated mitophagy outside the liver but cannot be extrapolated directly to steatotic hepatocytes. Future hepatic studies should use mitochondrial-specific flux reporters, such as mito-QC or mt-Keima, together with measurements of PINK1 stabilization, Parkin recruitment, phospho-ubiquitin, lysosomal delivery, and mitochondrial turnover.

The effects of BBR on autophagy may also be cell-type and disease-context dependent. In hepatic stellate cells and chemically induced mouse models of fibrosis, Yi et al. reported that BBR impaired the autophagy–lysosome pathway, increased ROS and redox-active iron accumulation, and promoted ferroptotic elimination of hepatic stellate cells [51]. Ferrostatin-1 attenuated the antifibrotic effects of BBR, supporting a functional contribution of ferroptosis. However, these were chemically induced fibrosis models rather than MASH-associated fibrosis. The findings therefore contrast with autophagy activation in lipid-loaded hepatocytes but do not establish how BBR regulates autophagy or ferroptosis in MASH-associated stellate cells. Collectively, these observations suggest that BBR is better characterized as a context-dependent regulator of hepatic quality-control pathways rather than as a universal activator of autophagy or mitophagy.

## 7. Membrane Potential, Proton Leak, and UCP2: Context-Dependent Effects

The mitochondrial membrane potential (ΔΨm) is generated by proton pumping through the respiratory chain and contributes to ATP synthesis, mitochondrial protein import, calcium uptake, and metabolite transport. Marked depolarization and sustained hyperpolarization of ΔΨm can be maladaptive: depolarization impairs ATP synthesis and may initiate mitophagy or cell-death signaling, whereas excessive polarization can increase respiratory-chain reduction and ROS generation. Consequently, an increase in ΔΨm should not automatically be interpreted as improved mitochondrial health without complementary measurements of respiration, ATP synthesis, proton leak, and ROS production [45,52,53]. Maintenance of ΔΨm within an appropriate range, rather than its maximal elevation, is required for mitochondrial function.

In HFD-fed rats with established hepatic mitochondrial dysfunction, Teodoro et al. reported that BBR restored ΔΨm, oxygen consumption, respiratory control, cellular ATP levels, and mitochondrial calcium-retention capacity [33]. These findings provide direct functional evidence that BBR can improve hepatic mitochondrial bioenergetics when oxidative phosphorylation is impaired. Increased SIRT3 activity accompanied these improvements, although the study did not use SIRT3 inhibition or deletion and therefore did not establish a causal role for SIRT3.

In contrast, BBR did not uniformly preserve ΔΨm or ATP availability in an oleic-acid-loaded HepG2 model. Rafiei et al. showed that 10 µM BBR reduced steatosis and increased SIRT1 activity and AMPKα phosphorylation but did not restore the oleic-acid-induced reduction in ΔΨm and further decreased cellular ATP under galactose-supported conditions [21]. These findings support a context-dependent bioenergetic effect in which anti-steatotic activity can occur despite incomplete recovery of membrane potential and reduced mitochondria-dependent ATP availability. However, the transformed HepG2 model and the 10 µM exposure limit direct clinical extrapolation.

UCP2 has also been implicated in altered hepatic bioenergetics during fatty liver disease [45]. Increased UCP2 expression may influence mitochondrial coupling and ROS generation, but UCP2 should not be regarded simply as a classical proton uncoupler. Experimental studies have linked UCP2 to proton conductance under some conditions, whereas other evidence suggests that it transports C4 metabolites and thereby modulates mitochondrial substrate oxidation. Its physiological contribution to hepatic proton leak and energy conservation therefore remains context dependent and incompletely defined [54,55].

In HFD-fed rats, Yang et al. reported that hepatic UCP2 mRNA and immunoreactive protein were increased by the diet and reduced by oral BBR at 162 or 324 mg/kg/day for 12 weeks [56]. These changes occurred alongside improvements in hepatic steatosis and serum and liver lipid concentrations. In a subsequent HFD-fed rat study, BBR at 100 mg/kg/day for 16 weeks increased hepatic SIRT1 expression, reduced UCP2 expression, and improved hepatic lipid accumulation and oxidative-stress markers [57]. However, the parallel changes in SIRT1 and UCP2 expression do not establish that SIRT1 directly mediated the reduction in UCP2.

These studies measured UCP2 expression but did not directly assess UCP2-dependent proton conductance, mitochondrial coupling efficiency, respiratory control, or ATP levels. Reduced UCP2 expression may therefore be secondary to attenuation of hepatic lipid overload or oxidative stress rather than a primary mechanism of BBR action. A cautious interpretation is that BBR normalizes diet-associated UCP2 overexpression in some fatty-liver models, whereas the consequences for proton leak, ROS generation, substrate transport, and mitochondrial energy efficiency remain unresolved.

Overall, BBR exerts model-dependent effects on membrane potential and respiratory coupling. It can restore ΔΨm, respiratory control, and cellular ATP levels in animals with established hepatic mitochondrial dysfunction, yet it can also reduce complex I-linked respiration and ATP availability in models characterized by nutrient or substrate overload. These observations are not necessarily contradictory: improved respiratory efficiency in damaged mitochondria may coexist with reduced substrate-driven respiratory activity in other metabolic states. Future studies should assess ΔΨm together with state-specific oxygen consumption, ATP-linked respiration, proton leak, respiratory-control ratios, and mitochondrial ROS or hydrogen-peroxide emission to determine whether BBR improves coupling, constrains respiratory demand, or produces both effects.

## 8. Mitochondrial Redox Remodeling and Antioxidant Defense

Mitochondrial ROS are generated when electrons leak from redox centers within the respiratory chain and reduce molecular oxygen, particularly under conditions of excessive substrate supply, altered membrane potential, respiratory-chain dysfunction, or insufficient antioxidant capacity [45,58]. At physiological concentrations, mitochondrial ROS participate in redox signaling; however, sustained overproduction can damage mitochondrial DNA, proteins, and membrane lipids, impair respiratory function, activate stress-sensitive inflammatory pathways, and promote hepatocyte injury [58,59]. Oxidative stress in MASLD therefore reflects an imbalance between ROS generation and antioxidant removal rather than the mere presence of ROS [45,58]. Persistent substrate excess and mitochondrial respiratory inefficiency are linked to increased mitochondrial ROS production, lipid peroxidation, inflammatory signaling, and hepatocyte injury in MASLD [45,58].

BBR may reduce oxidative stress through both mitochondrial and nonmitochondrial mechanisms. Sun et al. found that BBR reduced hepatic triglyceride accumulation in HFD-fed mice. In fatty-acid-exposed Huh7 cells, BBR suppressed NOX2 expression, NOX2-dependent cellular ROS, mitochondrial superoxide, and the expression of several respiratory-chain complexes; in HepG2 cells, it also reduced basal oxygen consumption [60]. These findings support respiratory suppression accompanied by reduced ROS generation; however, electron leakage and respiratory-chain enzymatic activity were not directly measured. The results should therefore not be interpreted as evidence of restored respiratory-chain expression or function.

The Nrf2 findings across BBR studies are model dependent. Sun et al. observed reduced HFD- or fatty-acid-associated Nrf2 nuclear localization and lower HO-1 expression following BBR treatment, which the authors attributed to attenuation of the oxidative stimulus that had activated the compensatory Nrf2 response [60]. In contrast, Deng et al. reported that BBR increased hepatic Nrf2, HO-1, and NQO1 mRNA and protein expression in HFD-fed rats [26]. BBR also increased hepatic superoxide dismutase and glutathione levels while reducing malondialdehyde. However, Deng et al. did not directly measure Nrf2 nuclear translocation, ARE binding, or transcriptional reporter activity. Taken together, these findings do not support a uniform Nrf2-activating effect of BBR. Instead, the direction and magnitude of Nrf2-related responses appear to depend on disease severity, experimental timing, tissue context, and baseline redox status. In some models, BBR enhances Nrf2-associated antioxidant signaling, whereas in others, reduced Nrf2 activation may reflect attenuation of the underlying oxidative stimulus. Future studies should therefore evaluate Nrf2 nuclear localization or DNA-binding activity alongside target-gene transcription, ROS generation, and markers of oxidative damage.

SIRT3 provides a plausible additional link between BBR and mitochondrial antioxidant defense. As a mitochondrial NAD^+^-dependent deacetylase, SIRT3 can activate antioxidant enzymes, including manganese superoxide dismutase (MnSOD), through deacetylation [61,62]. Deacetylation of MnSOD at Lys122 has been shown to increase its activity and reduce mitochondrial superoxide [61]. Teodoro et al. associated increased hepatic mitochondrial SIRT3 activity with improved respiratory control and ATP status following BBR treatment [33], whereas Xu et al. demonstrated SIRT3-dependent metabolic effects involving LCAD deacetylation [43]. However, neither study directly established that BBR reduced mitochondrial ROS through SIRT3-mediated MnSOD deacetylation in mammalian MASLD. This mechanism should therefore be presented as biologically plausible rather than established.

Evidence from a multicellular human-derived in vitro model further supports the anti-steatotic and redox effects of BBR. In a triculture containing HepG2 hepatocytes, differentiated THP-1 macrophage-like cells, and LX-2 stellate cells, treatment with 5 µM BBR for 72 h reduced hepatocyte lipid accumulation and intracellular ROS, lowered MIP-1α, MIP-1β, MCP-1, and IL-7 levels, and prevented the activation-mixture-induced increase in collagen I production by stellate cells [20]. IL-6 and IL-8 were not significantly reduced, indicating selective rather than generalized suppression of inflammatory mediators. However, the study did not measure mitochondrial ROS, respiration, respiratory-chain activity, or mitochondrial antioxidant enzymes; therefore, the reduction in intracellular ROS cannot be attributed specifically to improved mitochondrial redox function.

Collectively, the redox effects of BBR appear to involve suppression of NOX2-dependent ROS production, reductions in mitochondrial superoxide and respiratory activity in some hepatoma-cell models, and model-dependent regulation of Nrf2-associated antioxidant defenses. These effects may help interrupt the feed-forward relationship among lipid overload, oxidative damage, inflammatory signaling, and hepatocellular injury. Nevertheless, future studies should distinguish total cellular ROS from mitochondrial ROS and avoid equating lower malondialdehyde levels or increased antioxidant-enzyme expression with direct restoration of mitochondrial redox function.

## 9. Mitochondria-Dependent Apoptosis and Cell-Fate Signaling

The intrinsic apoptotic pathway involves activation of BAX and BAK, mitochondrial outer-membrane permeabilization, cytochrome-c release, apoptosome formation, and caspase-9 and caspase-3 activation. Its regulation depends on interactions among proapoptotic effectors, BH3-only proteins, and antiapoptotic BCL-2-family proteins rather than on the BAX/BCL-2 ratio alone [63,64]. In MASH, sustained hepatocyte apoptosis may amplify inflammation and fibrogenesis through damage-associated signals and apoptotic bodies [64].

Direct evidence that BBR suppresses mitochondria-dependent apoptosis specifically in mammalian MASLD models remains less extensive than the evidence supporting its effects on lipid metabolism, insulin signaling, and oxidative stress. In L02 hepatocyte cells and a D-galactosamine/lipopolysaccharide-induced mouse model of acute liver failure, BBR reduced the BAX/BCL-2 ratio, cytosolic cytochrome-c accumulation, and cleavage of caspases-9 and -3, together with attenuation of NF-κB-associated inflammatory signaling [65]. These findings support inhibition of mitochondria-associated apoptosis in acute inflammatory liver injury. However, acute liver failure differs substantially from chronic metabolically driven steatotic liver disease. Therefore, this study should be interpreted as supportive, non-MASLD evidence rather than direct evidence for a mechanism in MASLD.

In db/db mice with fatty liver, BBR reduced hepatic lipid accumulation, oxidative stress, inflammatory-marker expression, and TUNEL-positive hepatocyte death while increasing AMPK- and SIRT1-associated signaling [66]. These findings are consistent with an AMPK/SIRT1-associated reduction in hepatic apoptosis. Nevertheless, because the study did not employ pharmacological pathway inhibition, hepatocyte-specific gene deletion, or genetic disruption of AMPK or SIRT1, it does not establish that activation of this pathway was necessary or sufficient for the antiapoptotic effect of BBR.

Additional evidence derives from a nonmammalian fatty-liver model. In blunt snout bream fed an HFD, dietary BBR at 50 or 100 mg/kg diet for 8 weeks reduced hepatic oxidative stress and hepatocyte apoptosis, increased the activities of mitochondrial respiratory-chain complexes, and improved mitochondrial ultrastructure [67]. These findings support an association among preservation of mitochondrial function, reduced oxidative injury, and lower hepatocyte apoptosis. However, the aquatic species, dietary rather than body-weight-based dosing, and differences in hepatic lipid metabolism limit direct extrapolation to mammalian MASLD.

BBR-mediated regulation of mitochondrial architecture may also influence susceptibility to cellular injury. Xu et al. showed that BBR increased SIRT1 and OPA1 expression, reduced DRP1 expression, and improved mitochondrial morphology, membrane potential, ATP content, and insulin signaling in db/db mice and palmitate-treated HepG2 cells [47]. HFD-fed mice were used principally to characterize disease-associated changes in SIRT1, OPA1, MFN1, DRP1, and mitochondrial morphology rather than to demonstrate all BBR-induced responses. The study further implicated OPA1-dependent mitochondrial dynamics in the improvement of hepatic insulin signaling. However, it did not directly assess BAX or BAK activation, mitochondrial outer-membrane permeabilization, cytosolic cytochrome-*c* release, apoptosome formation, or caspase-9 activation. Thus, the findings support improved mitochondrial integrity but do not demonstrate that suppression of DRP1-associated fragmentation directly prevented intrinsic apoptosis.

Related evidence was provided by Teodoro et al., who reported that BBR treatment increased the calcium-retention capacity of hepatic mitochondria isolated from HFD-fed rats [33]. Because mitochondrial calcium-retention capacity is a functional measure of the calcium load required to trigger permeability transition, this result indicates reduced susceptibility to calcium-induced mitochondrial permeability-transition-pore opening. The study also reported restoration of hepatic mitochondrial function and increased SIRT3-associated activity. Nevertheless, the molecular composition or regulation of the pore was not examined, and the requirement for SIRT3 was not tested using inhibition, knockdown, or genetic deletion. Consequently, a causal SIRT3-dependent effect of BBR on permeability-transition-pore regulation cannot be concluded.

The cell-type and concentration dependence of BBR are particularly important when interpreting apoptosis studies. In HepG2 hepatoma cells, exposure to 50 µM BBR induced DNA fragmentation, loss of mitochondrial membrane potential, cytochrome-*c* release, activation of caspases-8 and -3, PARP cleavage, and Fas activation, together with reduced expression of Bid and BCL-XL [68]. Caspase inhibitors attenuated these effects, supporting the involvement of both death-receptor-associated and mitochondria-associated caspase signaling. This represents an antitumor cytotoxic response at a relatively high BBR concentration and should not be cited as evidence that induction of apoptosis is beneficial in MASLD. HepG2 cells may exhibit anti-steatotic or antioxidant responses at lower BBR concentrations but undergo cytotoxicity and apoptosis at higher exposures, emphasizing the importance of concentration, exposure duration, cellular phenotype, and experimental context.

Overall, preclinical evidence indicates that BBR can reduce hepatocyte apoptosis in several nonmalignant models of metabolic or inflammatory liver injury, whereas it can induce mitochondria-associated apoptosis in malignant hepatocyte-derived cells at cytotoxic concentrations. In metabolically stressed hepatocytes, the available evidence is most consistent with an indirect anti-apoptotic effect in which BBR attenuates lipotoxicity, oxidative stress, inflammatory signaling, and mitochondrial dysfunction, thereby reducing activation of the intrinsic apoptotic pathway. However, direct evidence that BBR prevents BAX or BAK activation, mitochondrial outer-membrane permeabilization, apoptosome formation, or mitochondrial permeability-transition-pore opening specifically in mammalian MASLD remains limited. Studies using primary hepatocytes, diet-induced MASLD models, and pathway-specific genetic or pharmacological approaches are needed to establish causality.

## 10. Emerging Mitochondrial Mechanisms and Hypothesis-Generating Evidence Gaps

The following mechanisms have unequal support. Functional or target-level evidence from a non-MASLD system establishes a testable mechanism in that system, but remains hypothesis-generating for BBR-treated mammalian MASLD unless disease-specific validation is available. MAM architecture, mitochondrial calcium uptake, mtDNA release, STING signaling, and ferroptotic death are distinct endpoints and should not be treated as interchangeable.

### 10.1. Mitochondria–Endoplasmic Reticulum (ER) Contact Sites and Calcium Handling

Mitochondria–ER contact sites are specialized interfaces at which the two organelles exchange lipids, calcium, and regulatory signals. Biochemically isolated ER membranes associated with mitochondria are commonly termed mitochondria-associated membranes (MAMs). These interfaces contribute to phospholipid synthesis and transfer, calcium signaling, mitochondrial fission, autophagosome formation, and cellular stress responses [69]. Important regulators include the IP3R–GRP75–VDAC1 calcium-transfer complex, the mitochondrial calcium uniporter, mitofusins, PACS2, and sigma-1 receptor. The IP3R–GRP75–VDAC1 complex facilitates calcium transfer from the ER to the mitochondrial intermembrane space, after which calcium enters the matrix through the mitochondrial calcium uniporter [70,71]. Physiological mitochondrial calcium uptake supports tricarboxylic-acid-cycle activity and oxidative phosphorylation, whereas excessive calcium transfer can promote mitochondrial calcium overload, ROS generation, permeability transition, and cell death [71,72].

BBR has been reported to attenuate ER stress and mitochondrial dysfunction in fatty-liver models, and Teodoro et al. showed that it increased the calcium-retention capacity of isolated hepatic mitochondria [33]. However, this finding does not establish that BBR regulates mitochondria–ER communication. The hepatic studies discussed here do not establish BBR-induced changes in contact-site abundance or spacing, IP3R–GRP75–VDAC1 assembly, or real-time ER-to-mitochondrial calcium flux in steatotic hepatocytes. Mitochondria–ER contact-site remodeling should therefore be presented as a mechanistic hypothesis rather than an established pathway of BBR action.

Zhao et al. identified BBR binding to mitochondrial calcium uniporter (MCU) and disruption of MCU–EMRE assembly, with inhibition of mitochondrial calcium uptake in cellular and cardiac-injury experiments [35]. These are direct functional and target-level observations in the tested systems, but do not establish MAM remodeling or benefit in steatotic human liver.

Future studies should combine contact-site imaging or proximity-ligation assays with genetically encoded calcium indicators targeted to the ER, cytosol, and mitochondrial matrix. Such approaches could determine whether BBR attenuates pathological mitochondrial calcium overload while preserving the physiological calcium signals required for oxidative metabolism.

### 10.2. Mitochondrial DNA Damage and Innate Danger Signaling

Persistent mitochondrial oxidative stress can damage mtDNA and promote its release into the cytosol or extracellular space. Cytosolic mtDNA can activate cGAS–STING signaling, whereas endosomal recognition of extracellular or internalized mtDNA may engage TLR9. Mitochondrial dysfunction and oxidized mtDNA can also contribute to NLRP3 inflammasome activation. Together, these pathways provide potential links between hepatocyte mitochondrial injury and sterile inflammatory signaling in liver macrophages [73,74,75].

Evidence connecting cGAS–STING signaling to fatty-liver progression must be interpreted according to the source of the DNA. Donné et al. showed that nucleotide-pool imbalance caused replication stress and DNA damage in proliferating fatty-liver hepatocytes, with subsequent activation of cGAS–STING signaling [76]. This study supports a role for DNA-damage-driven cGAS–STING activation but did not establish mtDNA leakage as the activating signal.

More direct evidence of a mtDNA-dependent pathway was provided by Miao et al. In HFD-related experimental models, selenoprotein W promoted PKM2–HIF-1α-associated metabolic reprogramming, mitochondrial damage, mitochondrial ROS accumulation, hepatocyte apoptosis and pyroptosis, and mtDNA release. Released mtDNA subsequently activated cGAS–STING signaling in macrophages and altered macrophage phenotype [77]. Although this study supports hepatocyte-to-macrophage mtDNA signaling in fatty-liver progression, it did not investigate BBR.

Because BBR reduces mitochondrial ROS and attenuates mitochondrial injury in some models, suppression of mtDNA damage and release is biologically plausible. However, evidence for altered STING signaling should be separated from evidence that mtDNA is the initiating ligand. Ji et al. reported PI3K/Akt-associated suppression of STING signaling in HFD-fed mice and palmitate-treated HepG2 cells while AKT inhibition attenuated the metabolic response [78]. This provides BBR-specific MASLD evidence at the signaling level, but does not establish that prevention of mtDNA oxidation or release mediates the response. Direct measurements and source-specific perturbations of mtDNA release remain necessary before assigning that causal mechanism.

### 10.3. Ferroptosis and Lipid-Peroxidation Stress

Ferroptosis is an iron-dependent form of regulated cell death characterized by the accumulation of peroxidized phospholipids and failure of lipid-antioxidant defenses, particularly the glutathione–GPX4 system [79]. Mitochondrial metabolism can modify ferroptotic sensitivity through context-dependent effects on ROS production, glutaminolysis, iron metabolism, and the availability of lipid-peroxidation substrates [80]. Ferroptotic hepatocyte injury has been implicated in MASH progression [81], but the effects of BBR are likely to depend on cell type and disease context [51].

In activated hepatic stellate cells and TAA- or CCl4-induced mouse models of fibrosis, Yi et al. reported that BBR impaired autophagy–lysosomal function, increased redox-active iron and ROS, depleted glutathione, enhanced lipid peroxidation, and promoted stellate-cell ferroptosis [51]. Ferrostatin-1 attenuated the antifibrotic effect of BBR, supporting a functional contribution of ferroptosis. However, these were chemically induced fibrosis models rather than metabolic MASH, and the results should not be extrapolated directly to steatotic hepatocytes.

Gu et al. also reported inhibition of ferroptosis in a study of NAFLD-associated bone loss [82]. The bone-focused ferroptosis findings do not establish protection against hepatocyte ferroptosis, even though hepatic steatosis also improved. This distinction prevents a disease label from being mistaken for evidence in the relevant hepatic cell population.

These findings indicate that BBR can promote oxidative injury and ferroptotic death in activated fibrogenic cells while reducing oxidative stress in hepatocyte models. At present, it remains unclear whether BBR protects steatotic hepatocytes from ferroptosis, promotes selective ferroptosis of activated stellate cells, or exerts both effects in a cell-specific manner during MASH. Future studies should combine measurements of lipid-peroxidation and iron status with assessment of GPX4/glutathione together with pharmacological rescue experiments in hepatocytes and stellate cells evaluated separately.

### 10.4. Additional Understudied Mechanisms

Several mitochondrial processes relevant to MASLD have received little or no direct investigation in relation to BBR. These include the mitochondrial unfolded protein response, mitochondrial proteases and chaperones, respiratory-chain supercomplex organization, cardiolipin remodeling and oxidation, mitochondrial translation, mitochondrial-derived vesicles, and mitochondrial-derived peptides. The effects of BBR on mitochondrial NAD^+^ availability, one-carbon metabolism, tricarboxylic-acid-cycle intermediates, and mitochondria-derived signaling metabolites also remain incompletely characterized. These mechanisms represent important research priorities, but mechanistic attribution should be avoided until they are directly evaluated in relevant BBR-treated MASLD models.

## 11. Summary of Preclinical Evidence

Preclinical studies have provided important insights into the effects of berberine on hepatic mitochondrial function and metabolic regulation in experimental models of MASLD. As summarized in Table 1, these studies report changes in mitochondrial respiration, ATP production, fatty acid oxidation, oxidative stress, mitochondrial dynamics, and autophagy. However, the effects appear context-dependent, with some studies demonstrating improvements in mitochondrial bioenergetics and others reporting respiratory suppression and reduced ATP availability. Differences in experimental models, dosing regimens, and outcome measurements further highlight the need for cautious interpretation and limit direct extrapolation to human MASLD.

**Table 1:** Representative preclinical studies evaluating the effects of berberine on hepatic mitochondrial and mitochondria-related outcomes.

Study	Model and Exposure	BBR Intervention	Principal Mitochondrial Findings	Critical Interpretation or Limitation
Teodoro et al., 2013 [33]	HFD-fed rats	100 mg/kg/day for 4 weeks	Restored hepatic mitochondrial ΔΨm, oxygen consumption, respiratory control, calcium-retention capacity, and cellular ATP levels; increased mitochondrial SIRT3 activity	Direct functional mitochondrial measurements; SIRT3 activation was associated with, but not proven necessary for, the bioenergetic improvements
Xu et al., 2019 [43]	HFD-fed wild-type and *Sirt3*-knockout mice	Oral BBR, approximately 140 mg/kg/day for 4 weeks	Increased SIRT3 expression, reduced LCAD hyperacetylation, and shifted acylcarnitine profiles in a direction consistent with improved β-oxidative metabolism	Sirt3 deletion markedly attenuated the metabolic effects and partially abolished the β-oxidation-related response; acylcarnitines are indirect markers rather than isotope-based flux measurements
Zhang et al., 2019 [25]	HFD-fed rats assessed after 8 and 16 weeks	BBR administered intragastrically at 100 mg/kg/day, concurrently with the HFD, for 8 or 16 weeks	Increased SIRT3 expression, p-AMPK, inhibitory p-ACC, and CPT1A protein abundance	Primarily protein-expression and phosphorylation evidence; no pathway disruption or direct mitochondrial respiration measurements
Wang et al., 2023 [44]	HFD-fed mice, primary hepatocytes, HepG2 and HEK293T mechanistic systems	Oral BBR in mice; BBR in lipid-loaded cells	Promoted SIRT1-mediated deacetylation of CPT1A at Lys675, reduced CPT1A ubiquitin-dependent degradation, increased CPT1A stability and activity, and promoted fatty-acid oxidation	Strong mechanistic evidence for post-translational regulation of CPT1A; not evidence of mitochondrial biogenesis or global improvement in mitochondrial respiratory function
Yu et al., 2021 [32]	C57BL/6J mice fed HFD for 20 weeks and oleate-treated primary hepatocytes	HFD containing 1.4 g BBR/kg diet	Reduced complex I-linked hepatic and intestinal oxygen consumption, reduced ATP-related bioenergetic output, suppressed hepatic fatty-acid uptake and β-oxidation, reduced mtDNA copy number and citrate synthase activity, increased mitochondrial length, and attenuated swelling	Supports complex I restraint and normalization of HFD-associated mitochondrial content-related indices rather than stimulation of biogenesis; 1.4 g/kg is a dietary concentration, not a body-weight dose
Xu et al., 2022 [47]	HFD-fed mice, db/db mice, and palmitate-treated HepG2 cells	BBR treatment in db/db mice and palmitate-treated HepG2 cells	Increased SIRT1 and OPA1, reduced DRP1, improved mitochondrial morphology, ATP content, and ΔΨm; SIRT1 silencing partly reversed cellular effects	Strongest intervention evidence was obtained in db/db mice and HepG2 cells. HFD-fed mice were used mainly for model characterization; direct fusion rates, mitophagic flux, and in vivo pathway causality were not measured
Sun et al., 2018 [48]	HFHS-fed wild-type and liver-specific *Sirt1*-knockout mice; primary hepatocytes and HepG2 cells	BBR 100 mg/kg by intraperitoneal injection for 5 weeks	Induced hepatic SIRT1-dependent autophagy, increased FGF21 and ketone-body responses, reduced steatosis, and increased whole-body energy expenditure	Strong evidence for hepatic SIRT1-dependent general autophagy, not selective mitophagy; intraperitoneal administration limits translation to oral supplementation
Sun et al., 2017 [60]	HFD-fed mice and rats; fatty-acid-treated Huh7 cells and HepG2 cells	Oral BBR in rodents and 10 µM BBR in cell models	Reduced NOX2-dependent cellular ROS, mitochondrial superoxide, respiratory-chain protein expression, and basal oxygen consumption	Reduced ROS was accompanied by respiratory suppression; respiratory-chain enzymatic activity and electron leakage were not directly measured, and Nrf2 responses differed from those in other models
Yang et al., 2011 [56]	HFD-fed rats	162 or 324 mg/kg for 12 weeks	Reduced hepatic UCP2 mRNA and immunoreactive protein alongside improved steatosis and lipid profiles	Did not assess UCP2-dependent proton conductance, coupling efficiency, respiration, or ATP synthesis
Rafiei et al., 2023 [20]	HepG2/LX-2/differentiated THP-1 human-derived triculture exposed to an activating mixture	5 µM for 72 h	Reduced hepatocyte steatosis and DCFH-DA-detected intracellular ROS, lowered selected cytokines and chemokines, and prevented the increase in stellate-cell collagen I	Multicellular human-derived model, but mitochondrial ROS, respiration, respiratory-chain activity, and mitochondrial antioxidant enzymes were not measured
Rafiei et al., 2019 [21]	HepG2 hepatocytes exposed to 1.5 mM oleic acid for 24 h	10 µM BBR administered during oleic-acid exposure	Reduced steatosis and increased SIRT1 activity and AMPK phosphorylation but did not restore ΔΨm and further reduced ATP content under galactose-supported conditions	Supports context-dependent energetic reprogramming rather than uniform mitochondrial enhancement; transformed HepG2 cells and 10 µM exposure limit clinical extrapolation
Rafiei et al., 2018 [24]	Palmitate-exposed HepG2 cells	10 µM during palmitate exposure	Reduced palmitate-induced lipid accumulation and intracellular ROS but did not substantially attenuate the increase in iNOS expression	Supports anti-steatotic and antioxidant effects but indicates incomplete protection against nitrosative-stress signaling; mitochondrial effects should be described only where BBR-specific data were reported
Rafiei et al., 2017 [23]	Oleic-acid-exposed HepG2 cells	BBR included among compounds tested at 1–10 µM	Reduced oleic-acid-induced cellular ROS; mitochondria-related markers were also evaluated across the intervention panel	BBR-specific effects should be distinguished from findings reported collectively across compound classes; mitochondrial respiration, ATP synthesis, and biogenesis were not directly demonstrated
Lu et al., 2017 [67]	Blunt snout bream fed a HFD	50 or 100 mg/kg of diet for 8 weeks	Increased respiratory-chain complex activities, improved mitochondrial ultrastructure, and reduced hepatic oxidative stress and apoptosis	Nonmammalian model and dietary concentration limit direct extrapolation to mammalian or human MASLD

Abbreviations: BBR, berberine; HFD, high-fat diet; HFHS, high-fat/high-sucrose diet; ΔΨm, mitochondrial membrane potential; ATP, adenosine triphosphate; SIRT1/3, sirtuins 1 and 3; LCAD, long-chain acyl-CoA dehydrogenase; AMPK, AMP-activated protein kinase; ACC, acetyl-CoA carboxylase; CPT1A, carnitine palmitoyltransferase 1A; p-, phosphorylated; mtDNA, mitochondrial DNA; TEM, transmission electron microscopy; OPA1, optic atrophy 1; DRP1, dynamin-related protein 1; FGF21, fibroblast growth factor 21; NOX2, NADPH oxidase 2; ROS, reactive oxygen species; Nrf2, nuclear factor erythroid 2-related factor 2; UCP2, uncoupling protein 2; DCFH-DA, dichlorodihydrofluorescein diacetate; iNOS, inducible nitric oxide synthase.

## 12. Human Clinical Evidence and Mitochondrial Outcomes

Clinical studies suggest that BBR-containing interventions may reduce liver fat and improve selected cardiometabolic outcomes; however, none has directly demonstrated restoration of hepatic mitochondrial function in humans. Improvements in aminotransferases, HOMA-IR, circulating lipids, body weight, or MRI-measured liver fat should therefore not be interpreted as direct evidence of mitochondrial improvement. These are intermediate imaging or metabolic outcomes. The included studies do not establish histologic MASH resolution or fibrosis improvement using biopsy-based endpoints, or reductions in cirrhosis progression, decompensation, transplantation, or liver-related mortality. Clinical metabolic effects can be evaluated in these trials, but their mechanistic implications remain hypothesis-generating and disease-modifying liver efficacy is unproven.

**Table 2:** Human clinical evidence for pure BBR and BBR-containing interventions in MASLD and assessment of direct mitochondrial outcomes.

Study	Design and Population	Intervention	Main Reported Outcomes	Interpretation	Product/Formulation	Safety and Tolerability
Yan et al., 2015 [27]	Multicenter, randomized, open-label, three-arm trial; 184 adults with NAFLD	Lifestyle intervention alone, lifestyle plus pioglitazone, or lifestyle plus BBR 0.5 g three times daily for 16 weeks	Greater reduction in hepatic fat content with BBR plus lifestyle than lifestyle alone; improvements in weight, HOMA-IR, and lipid profile	Supports clinical metabolic and liver-fat effects of pure BBR, but open-label design, concurrent lifestyle treatment, weight loss, and absence of mitochondrial measurements limit mechanistic inference	Purified BBR	Mainly mild digestive symptoms; one BBR withdrawal due to an adverse event [27].
Harrison et al., 2021 [29]	Phase 2, randomized, double-blind, placebo-controlled trial; 100 adults with presumed NASH and type 2 diabetes	BUDCA 500 or 1000 mg twice daily versus placebo for 18 weeks	The 1000 mg twice-daily dose reduced MRI-PDFF liver fat and improved glycemic control, liver-associated enzymes, and body weight	Distinct BBR–UDCA salt; effects cannot be assigned to pure BBR.	HTD1801/BUDCA ionic salt	Mainly gastrointestinal symptoms. AE withdrawals: placebo 1/33; 500 mg twice daily 1/33; 1000 mg twice daily 8/34. Not pure-BBR safety evidence.
Nie et al., 2024 [28]	Systematic review and meta-analysis of 10 RCTs involving 811 participants	Various BBR regimens, comparators, and treatment durations	Reported reductions in ALT, AST, GGT, triglycerides, total and LDL cholesterol, BMI, and HOMA-IR	Clinical findings are heterogeneous and do not validate a mitochondrial mechanism; individual trial quality and formulations should be evaluated carefully	BBR/add-on regimens plus HTD1801	Five of ten RCTs reported adverse events, mainly gastrointestinal; incomplete reporting limits inference.
Lei et al., 2026 [30]	Multicenter, double-blind, placebo-controlled randomized trial; 337 adults with obesity and MASLD without diabetes	Pure oral BBR, 1 g/day, or placebo for 6 months	No significant reduction in liver fat or visceral adipose tissue; exploratory reductions in LDL cholesterol, apolipoprotein B, and hs-CRP	Neutral evidence for liver-fat reduction with pure BBR in this population; no mitochondrial mechanism was assessed	Purified BBR HCl	BBR vs. placebo: serious AEs 3.6% vs. 1.2%; nonserious AEs of interest 10.1% vs. 7.7%; discontinuation 3.0% vs. 3.6%; no significant differences. A 30-day active-BBR run-in excluded intolerance and poor adherence.

Table 2. No included intervention trial measured a direct hepatic mitochondrial endpoint and pooled analyses do not supply such measurements. BBR, berberine; HCl, hydrochloride; BUDCA, berberine ursodeoxycholate; AE, adverse event. Product identity and safety must be interpreted separately from intermediate efficacy outcomes. ALT, alanine aminotransferase; AST, aspartate aminotransferase; GGT, gamma-glutamyl transferase; HOMA-IR, homeostatic model assessment of insulin resistance; MRI-PDFF, magnetic resonance imaging proton-density fat fraction; LDL, low-density lipoprotein; BMI, body mass index; RCT, randomized controlled trial; hs-CRP, high-sensitivity C-reactive protein; UDCA, ursodeoxycholic acid.

**Figure 2:**
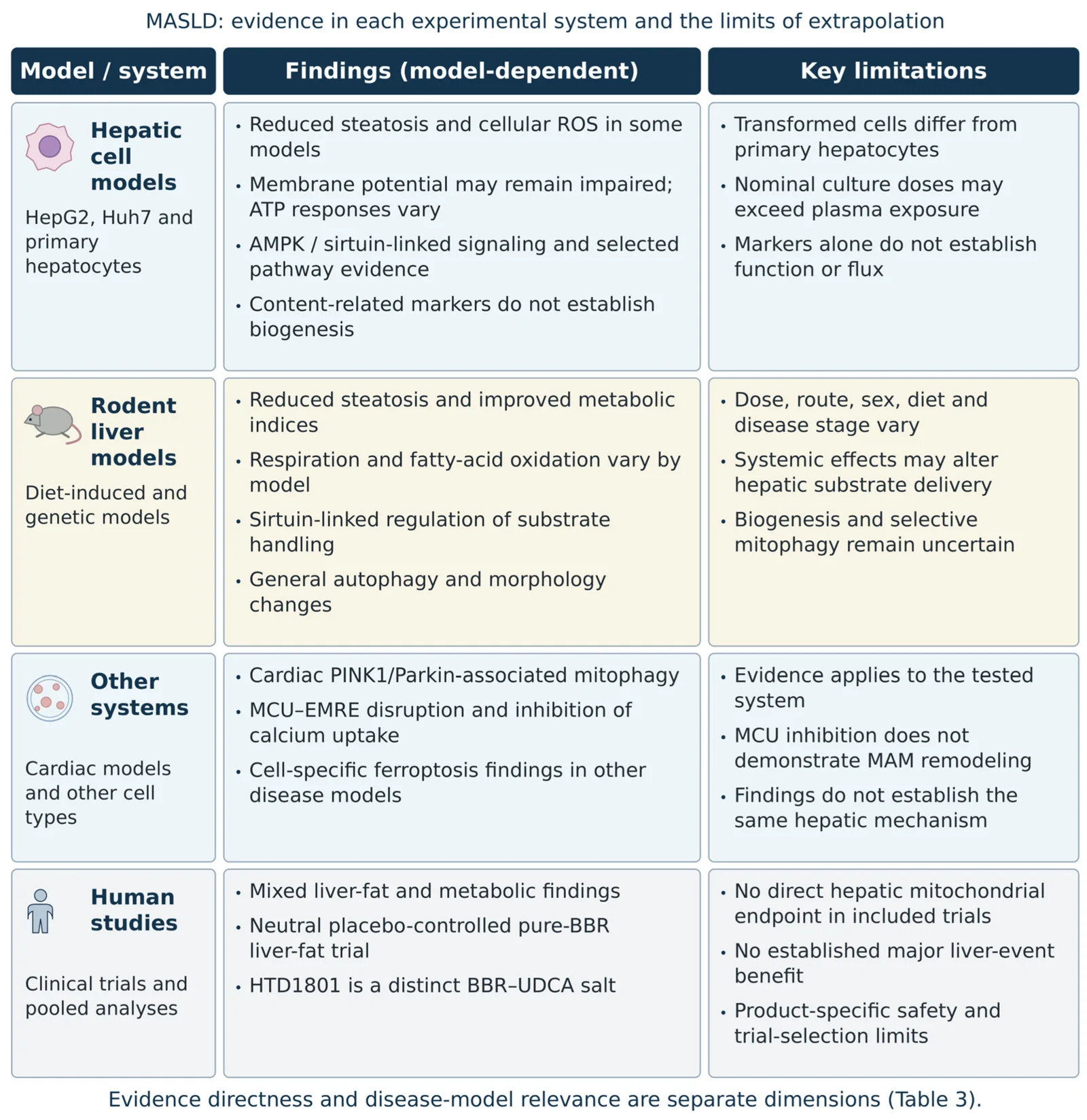
Translational evidence map for berberine-associated mitochondrial reprogramming in MASLD. Findings and interpretive limits are summarized by experimental system. Evidence directness and model relevance are separate dimensions, as defined in Table 3. Respiratory responses and biogenesis-associated markers vary across studies; neither increased biogenesis nor selective mitophagy is established as a consistent MASLD mechanism. Human findings are mixed and include a neutral pure-BBR liver-fat trial. No included trial directly assessed hepatic mitochondrial function or established a reduction in major liver-related events. Abbreviations: AMPK, AMP-activated protein kinase; ATP, adenosine triphosphate; BBR, berberine; EMRE, essential MCU regulator; MAM, mitochondria-associated membrane; MASLD, metabolic dysfunction-associated steatotic liver disease; MCU, mitochondrial calcium uniporter; PINK1, PTEN-induced kinase 1; ROS, reactive oxygen species; UDCA, ursodeoxycholic acid.

The human studies included in this review have not directly assessed hepatic mitochondrial respiration, ATP synthesis, respiratory coupling, membrane potential, mtDNA integrity or release, mitochondrial dynamics, mitochondrial protein acetylation, or mitophagic flux. Consequently, the current proposed mitochondrial mechanisms of BBR in humans remain inferential and are derived predominantly from preclinical models. The distribution of evidence across experimental systems and the major translational limitations of the current literature are summarized in Figure 2.

## 13. Discussion

The principal conclusion of this review is that BBR should not be characterized simply as either an inhibitor or an enhancer of mitochondrial function. Rather, the available evidence supports a context-dependent model of mitochondrial metabolic reprogramming [32,33,36,60]. BBR can directly restrict complex I-linked respiration and reduce ATP availability, thereby constraining mitochondrial substrate oxidation and cellular energy supply [21,60]. Under other experimental conditions, particularly in mitochondria impaired by metabolic stress, BBR has been associated with restoration of membrane potential, respiratory control, cellular ATP levels, and calcium-retention capacity [33,47,67]. These findings appear contradictory only if mitochondrial health is equated with maximal respiratory activity. In metabolically overloaded liver, both excessive substrate-driven electron flux and inadequate ATP-generating capacity may contribute to dysfunction [11,45].

Within this framework, BBR may impose a moderate energetic constraint while engaging compensatory metabolic and quality-control responses [48,49]. Partial complex I inhibition can alter cellular energy status and activate AMPK-associated signaling [36,83], whereas SIRT1 and SIRT3 can modify transcriptional programs and the acetylation state of metabolic proteins [33,43,44]. The SIRT1–CPT1A and SIRT3–LCAD pathways provide particularly informative examples because they link BBR to post-translational regulation of mitochondrial fatty-acid entry and oxidation [43,44].

Nevertheless, the direction of change in fatty-acid oxidation differs among models. In models characterized by impaired β-oxidation, BBR increased molecular or metabolomic indices consistent with improved fatty-acid oxidation [25,43,44], whereas in other models it reduced fatty-acid uptake and oxidative metabolism [32]. The therapeutic objective may therefore be restoration of substrate–demand matching and metabolic flexibility rather than maximal fatty-acid oxidation [45,84].

BBR also influences mitochondrial architecture, autophagy, redox regulation, and susceptibility to cell death [47,48,49,60]. Increased SIRT1–OPA1-associated signaling and reduced DRP1 expression have been accompanied by less fragmented mitochondrial morphology and improved insulin signaling [47]. SIRT1-dependent and ATG5-associated autophagy contributes to reduced hepatic lipid accumulation, although direct evidence for selective mitophagy in MASLD remains limited [48,49].

Likewise, BBR reduces mitochondrial superoxide and oxidative injury through NOX2 suppression, respiratory remodeling, and model-dependent antioxidant responses [26,33,60]. However, lower ROS should not automatically be interpreted as evidence of increased respiratory-chain function. In several studies, reductions in ROS occurred alongside decreased oxygen consumption, ATP availability, or respiratory-chain protein expression [21,32,60].

Studies by Rafiei et al. further illustrate the need to distinguish anti-steatotic efficacy, mitochondrial signaling, and direct bioenergetic restoration. Across several fatty-acid-loaded HepG2 models, BBR reduced steatosis and cellular ROS and modified SIRT1–AMPK–PGC-1α signaling and mitochondrial content-related markers, but it did not consistently restore membrane potential and reduced ATP availability under galactose-supported conditions. In a human-derived triculture, BBR also reduced steatosis, intracellular ROS, selected inflammatory mediators, and collagen I without direct assessment of mitochondrial function [20,21,23,24].

**Table 3:** Operational classification of mitochondrial evidence used in this narrative review. Categories overlap and do not constitute a formal quality ranking. A study may contain causal pathway evidence and only indirect evidence of oxidation flux. Model relevance, assay controls, exposure, replication, and bias are assessed separately.

Category	Operational Criterion and Example	Interpretive Limit
Direct functional	Substrate-defined respiration, ATP-production rate, coupling/proton leak, controlled ΔΨm or calcium uptake; e.g., isolated-liver mitochondrial assays [32,33].	Describes the measured function in the tested model. ATP content is not production rate; clinical biomarkers are not direct hepatic mitochondrial assays.
Mechanistic/causal	Genetic disruption, target interrogation, pathway inhibition or rescue; e.g., Sirt3 knockout, liver-specific Sirt1 deletion, CPT1A-site interrogation [43,44,48].	Tests dependence or target engagement, not necessarily sufficiency. Off-target inhibition and systemic knockout effects require qualification.
Indirect/supportive	Expression, phosphorylation, acetylation, static morphology, mtDNA copy number, acylcarnitines, or clinical imaging/biochemistry.	Supports pathway engagement or phenotype; does not by itself establish biogenesis, fusion/fission rate, mitophagic flux, or β-oxidation flux.
Hypothesis-generating	Extrapolation from nonhepatic/non-MASLD systems or incompletely tested disease-specific links; e.g., MCU targeting as a candidate MASLD mechanism [35].	Biological plausibility or proof in another model is not proof of the same causal mechanism in MASLD.

### 13.1. Strengths and Limitations of the Mechanistic Evidence

As summarized in Figure 2, mechanistic evidence is strongest in hepatocyte and rodent models and substantially weaker at the human clinical level, where direct mitochondrial endpoints are lacking. The most informative evidence for specific causal mechanisms comes from studies employing genetic disruption, pathway inhibition, or functional rescue. Attenuation of BBR-associated metabolic effects in Sirt3-knockout mice supports a functional contribution of SIRT3 and implicates LCAD deacetylation [43]. Similarly, liver-specific Sirt1 deletion supports a role for SIRT1-dependent autophagy, whereas ATG5-focused experiments support the contribution of autophagic machinery to selected metabolic effects [48]. SIRT1 knockdown studies also support involvement of the SIRT1–CPT1A and SIRT1–OPA1 pathways [44,47]. Such approaches provide stronger mechanistic evidence than observations restricted to changes in protein abundance.

Despite these strengths, the evidence base has important limitations. Most studies have used male rodents, transformed hepatoma-cell lines, or nonmammalian species. HepG2 and Huh7 cells differ from primary hepatocytes in substrate metabolism, respiratory dependence, proliferative state, and mitochondrial phenotype. In vitro BBR concentrations may also exceed unbound concentrations achieved in human tissues after conventional oral supplementation. Animal doses, formulations, and administration routes vary widely, including oral gavage, dietary incorporation, and intraperitoneal injection, making comparisons of systemic and hepatic exposure difficult.

Several studies rely on surrogate markers rather than direct measurements of mitochondrial function. Increased PGC-1α expression is sometimes interpreted as mitochondrial biogenesis without confirmation using complementary measures such as mtDNA copy number, mitochondrial protein content, organelle mass, ultrastructure, or respiratory capacity. Similarly, LC3-II abundance is occasionally interpreted as evidence of mitophagy, while changes in DRP1 or OPA1 abundance are treated as direct measurements of mitochondrial fission or fusion. Acylcarnitine profiles may support altered fatty-acid metabolism but do not establish β-oxidation flux in the absence of isotope tracing or comparable functional approaches. These distinctions are essential for avoiding mechanistic overstatement.

In particular, an increase in mitochondrial content-related markers is not equivalent to an increased biogenesis rate, and steady-state mitochondrial abundance reflects both synthesis and clearance. Membrane-potential and fluorescent ROS assays are functional readouts only within their assay-specific controls, while neither establishes ATP synthesis or a specific ROS source by itself. Dynamic turnover or flux measurements are needed to distinguish pathway activity from static accumulation.

Whole-animal improvements may also arise indirectly through reduced food intake, reduced body-weight gain, altered intestinal lipid handling, bile-acid–FXR signaling, gut-microbiota remodeling, or adipose-tissue metabolic signaling [22,32,85]. Yu et al. provided evidence that complex I inhibition in both the intestine and liver contributed to altered lipid handling and increased fecal lipid loss [32]. Consequently, improvements in hepatic mitochondrial phenotype may partly reflect reduced substrate delivery rather than exclusively hepatocyte-autonomous effects. Pair-feeding, tissue-specific genetic models, pharmacokinetic measurements, and organ-targeted formulations will be important for distinguishing these mechanisms.

Available studies also provide limited information regarding biological sex, disease stage, genetic susceptibility, and fibrosis severity. Mitochondrial adaptations during early steatosis may differ substantially from those present in inflammatory MASH or advanced fibrosis [11,45,86]. The same BBR exposure could therefore suppress excessive respiratory activity during early nutrient overload yet be insufficient to restore severely damaged mitochondria in advanced disease; however, this remains a stage-dependent hypothesis requiring direct testing. Sex-stratified and disease-stage-specific studies are needed before a unified dose–response or stage-dependent model can be established.

### 13.2. Human Evidence and Translational Limitations

Clinical interpretation requires separation of product identity, endpoint type, and study design. Conventional purified BBR salts, multi-component BBR-containing products, and HTD1801 are not interchangeable interventions. Liver fat, aminotransferases, HOMA-IR/HbA1c, lipids, weight, and inflammatory biomarkers do not demonstrate restoration of hepatic mitochondrial function or reduced major liver-related clinical events. No included study establishes a mitochondria-mediated effect in humans.

Yan et al. provided early randomized clinical evidence that oral BBR, when combined with lifestyle intervention, could reduce hepatic fat content and improve selected metabolic outcomes in adults with NAFLD [27]. Participants receiving BBR plus lifestyle intervention experienced a greater relative reduction in hepatic fat content than those receiving lifestyle intervention alone, together with greater improvements in body weight, HOMA-IR, and serum lipid variables. However, the trial was open label, BBR was administered concurrently with lifestyle intervention, and the greater weight loss in the BBR group may have contributed to the reduction in hepatic fat. No direct mitochondrial outcome was assessed. These findings should be considered alongside more recent placebo-controlled evidence. In a randomized trial involving 337 adults with obesity and MASLD but without diabetes, treatment with pure BBR at 1 g/day for six months did not significantly reduce liver fat content or visceral adipose tissue compared with placebo, although modest reductions in LDL cholesterol, apolipoprotein B, and hs-CRP were observed [30]. Differences in population, metabolic phenotype, formulation, dose, duration, and background intervention may contribute to discordant trial results, but the neutral trial does not identify which factors explain those differences. Accordingly, the possibility of subgroup-specific efficacy remains a hypothesis rather than an established explanation [30].

The phase 2 trial by Harrison et al. demonstrated a greater reduction in MRI-PDFF-measured liver fat and improvements in glycemic control, liver-associated enzymes, and body weight with the higher dose of berberine ursodeoxycholate compared with placebo [29]. However, berberine ursodeoxycholate, also termed HTD1801, is an ionic salt of BBR and ursodeoxycholic acid and represents a distinct molecular entity rather than pure BBR. These findings therefore cannot establish the efficacy of conventional BBR supplements or attribute the observed effects specifically to BBR-mediated mitochondrial remodeling. Its efficacy and adverse-event profile cannot be assigned to BBR alone [29].

The systematic review and meta-analysis by Nie et al. included 10 randomized trials involving 811 participants and reported pooled reductions in ALT, AST, GGT, triglycerides, total cholesterol, LDL cholesterol, BMI, and HOMA-IR [28]. However, these biochemical and anthropometric outcomes do not validate the proposed mitochondrial mechanisms. Moreover, heterogeneity in BBR formulations, doses, diagnostic criteria, comparator treatments, intervention durations, and trial quality limits confidence in a unified clinical or mechanistic interpretation. The pooled trials include HTD1801 and BBR add-on regimens, so the pooled effects cannot be assigned specifically to pure BBR.

Safety and tolerability also require product-specific interpretation (Table 2). Yan et al. reported mainly mild digestive symptoms and one adverse-event-related BBR discontinuation [27]. Harrison et al. reported gastrointestinal adverse effects with HTD1801, particularly diarrhea and abdominal discomfort [29]. In Lei et al., serious adverse events occurred in 3.6% versus 1.2%, nonserious events of interest in 10.1% versus 7.7%, and treatment discontinuation in 3.0% versus 3.6% (BBR versus placebo), without statistically significant differences [30]. The trial included a 30-day active-BBR run-in that excluded participants with intolerance or poor adherence, which limits generalization of tolerability estimates. Absence of a significant difference does not establish safety equivalence or exclude rare or delayed harms. The meta-analysis reported adverse-event data in only five of ten trials, mainly gastrointestinal symptoms [28].

Potential drug interactions are clinically relevant in MASLD, where polypharmacy is common. In a human crossover study, BBR 300 mg three times daily for two weeks reduced CYP2D6, CYP2C9, and CYP3A4 activities [87]. Trials should therefore document concomitant medication, product purity, adverse-event ascertainment, severity, causality, and withdrawals rather than infer safety from nutraceutical status. Short-term tolerability in selected participants cannot establish long-term safety in advanced liver disease.

Translation is further complicated by the low and variable oral bioavailability of BBR. Limited intestinal absorption, efflux transport, extensive intestinal and hepatic first-pass metabolism, microbial biotransformation, and the formation of metabolites such as berberrubine, demethyleneberberine, jatrorrhizine, and columbamine can influence systemic exposure and biological activity [17,31,88]. Animal pharmacokinetic studies indicate that hepatic exposure may substantially exceed plasma exposure despite very low circulating BBR concentrations. However, comparable quantitative evidence in human liver remains limited, making direct comparison between oral supplementation and the micromolar concentrations used in cell-culture exposure problematic. Future clinical studies should characterize parent BBR and relevant metabolites in plasma and, where ethically and technically feasible, liver tissue.

Human pharmacokinetic data illustrate the exposure gap. In a randomized crossover study of 12 healthy adults, 800 mg BBR powder produced a parent-BBR plasma Cmax of 0.9 nM, while vitamin E TPGS and Quillaja emulsification increased Cmax to 2.0 and 5.1 nM, respectively, with berberrubine and demethyleneberberine as major phase-1 metabolites [89]. A nominal 10 µM culture concentration is approximately 11,000-fold above the 0.9 nM peak in that particular powder condition. This is an illustrative comparison, not an estimate of liver exposure or a universal bioavailability ratio. Plasma total/free concentration, protein binding, exposure time, intracellular accumulation, active metabolites, and formulation differ between models. Preferential hepatic distribution in rats [31] does not establish the same quantitative distribution in humans. Animal mg/kg doses, dietary concentrations, and intraperitoneal dosing cannot be converted into clinically equivalent exposures without pharmacokinetic data.

### 13.3. Unresolved Mechanisms

Several fundamental questions remain unanswered. First, it is not known whether mitochondrial complex I inhibition is necessary for the metabolic effects of BBR in mammalian MASLD or whether it represents only one of several dose-, tissue-, and context-dependent actions. Second, the intracellular and submitochondrial concentrations of BBR and its metabolites in hepatocytes have not been adequately characterized. Third, the relative contributions of intestinal complex I inhibition, altered intestinal lipid handling, gut-microbiota remodeling, bile-acid signaling, and BBR metabolites must be distinguished from direct hepatocyte-autonomous effects.

Fourth, evidence for BBR-induced mitochondrial biogenesis remains inconsistent. Increased PGC-1α expression may indicate activation of a biogenesis-associated transcriptional program but does not, by itself, demonstrate increased mitochondrial content or functional capacity. In contrast, Yu et al. reported that BBR reduced or normalized HFD-associated increases in mtDNA copy number and citrate synthase activity, supporting mitochondrial remodeling rather than uniform stimulation of biogenesis. Fifth, selective mitophagy has not been convincingly demonstrated in BBR-treated mammalian MASLD models, despite evidence that BBR can promote general autophagy. Sixth, the effects of BBR on mitochondria–ER contact sites, organelle calcium transfer, mtDNA damage and release, cGAS–STING signaling, hepatocyte ferroptosis, cardiolipin remodeling, respiratory-chain supercomplex organization, and mitochondrial proteostasis require further disease-specific validation. Recent MCU-target and STING-pathway studies [35,78] support selected components but do not establish MAM remodeling or an mtDNA-release-mediated mechanism in MASLD.

Finally, BBR may exert divergent effects across hepatic cell populations. It can reduce oxidative stress and apoptosis in metabolically stressed hepatocyte models while inducing apoptosis in hepatoma cells and ferroptosis in activated hepatic stellate cells depending on the experimental model and exposure conditions. Such cell-selective effects may have therapeutic relevance, but they preclude the assumption that BBR uniformly activates or suppresses a given pathway throughout the liver.

### 13.4. Research Priorities and Translational Roadmap

Future mechanistic studies should prioritize primary human hepatocytes, induced-pluripotent-stem-cell-derived hepatocyte-like cells, multicellular liver organoids, and precision-cut human liver slices. These systems should be compared directly with conventional hepatoma-cell models to determine whether the effects of BBR observed at micromolar concentrations are reproduced in metabolically competent human tissue.

Mitochondrial assessments should include high-resolution respirometry using defined substrates, inhibitors, and uncouplers; ATP content and ATP-production rate; respiratory-control and coupling indices; proton leak; membrane potential; and simultaneous assessment of mitochondrial ROS or hydrogen-peroxide generation. Stable-isotope tracers should be used to quantify fatty-acid uptake and oxidation, tricarboxylic-acid-cycle flux, ketogenesis, and de novo lipogenesis. Mitochondrial content and biogenesis should be evaluated using complementary measurements of mtDNA copy number, citrate synthase activity, mitochondrial protein abundance, organelle mass, ultrastructure, and respiratory capacity rather than PGC-1α expression alone.

Mitophagy should be assessed using mitochondria-specific flux reporters together with lysosomal inhibition, whereas mitochondrial fusion and fission should be evaluated through live-cell imaging, OPA1 processing, DRP1 localization and phosphorylation, and appropriate functional perturbations. ER-to-mitochondrial calcium transfer and contact-site architecture should be examined using organelle-targeted calcium indicators, proximity-based assays, and electron microscopy. Measurements of oxidized mtDNA, cytosolic and extracellular mtDNA, cGAS–STING–TBK1–IRF3 signaling, and cell-type-specific ferroptosis markers should also be incorporated.

Dose selection should be informed by pharmacokinetic and tissue-exposure data. Studies should report BBR purity, formulation, route of administration, parent-compound and metabolite concentrations, and, where feasible, subcellular or mitochondrial accumulation. Pair-fed and body-weight-matched control groups are needed to determine whether hepatic effects are independent of changes in energy intake or body weight. Tissue-specific knockout, pathway inhibition, and rescue experiments should be used to identify mechanisms that are necessary for BBR-associated effects rather than merely correlated with them.

Clinical trials should evaluate chemically characterized pure BBR separately from combination products and enhanced-bioavailability formulations. Prespecified mitochondria-related outcomes could include circulating acylcarnitine and organic-acid profiles, ketone kinetics, phosphorus magnetic-resonance spectroscopy, liver-tissue respiratory and biochemical measurements in biopsy substudies, and transcriptomic or proteomic signatures of mitochondrial pathways. Because circulating biomarkers and magnetic-resonance measures are indirect, they should complement rather than replace direct hepatic measurements. Nevertheless, their inclusion would represent a substantial advance over reliance on aminotransferases, lipid profiles, and HOMA-IR alone.

## 14. Conclusions

BBR exerts context-dependent effects on hepatic mitochondrial metabolism rather than uniformly stimulating or inhibiting mitochondrial activity. Its reported actions include partial inhibition of complex I-linked respiration, modulation of AMPK and sirtuin signaling, post-translational regulation of CPT1A and LCAD, remodeling of mitochondrial architecture, activation of autophagy, and attenuation of oxidative stress and apoptosis. The direction of its effects on respiration and fatty-acid oxidation varies with the experimental model, exposure conditions, substrate load, and baseline mitochondrial state, supporting its characterization as a mitochondrial metabolic reprogrammer rather than a conventional mitochondrial enhancer.

The strongest pathway-specific evidence supports SIRT3-associated LCAD deacetylation, SIRT1-mediated stabilization of CPT1A, SIRT1-dependent autophagy, and model-dependent respiratory-chain remodeling. In contrast, evidence for consistent mitochondrial biogenesis, selective mitophagy, regulation of mitochondria–ER contact sites, suppression of mtDNA-associated danger signaling, or protection against hepatocyte ferroptosis remains insufficient.

Human evidence remains limited and mixed, and no included clinical trial has directly measured hepatic mitochondrial function or demonstrated a mitochondria-mediated clinical benefit. Translation is constrained by variable formulations, bioavailability, supplement quality, study design, and the absence of mitochondria-focused clinical endpoints. BBR therefore remains a mechanistically promising candidate for mitochondrial metabolic remodeling in MASLD, but confirmation requires standardized interventions and well-controlled human studies incorporating direct or rigorously validated hepatic mitochondrial outcomes. Improvements in intermediate metabolic endpoints do not establish histologic benefit or reduced major liver-related events. Safety and efficacy remain product-, population-, and exposure-specific.

## Data Availability

No new data were created or analyzed in this study. Data sharing is not applicable to this article.
